# A Pulmonary Abscess Caused by *Porphyromonas gingivalis* Infection: A Case Report and Literature Review

**DOI:** 10.1111/crj.70099

**Published:** 2025-07-16

**Authors:** Xu Chen, Ling Wu, Ruoxi Wu, Jiajia Dong

**Affiliations:** ^1^ Department of Pulmonology Shanxi Provincial Hospital of Traditional Chinese Medicine Taiyuan Shanxi Province China; ^2^ Department of Respiratory and Critical Care Medicine The First People's Hospital of Shuangliu District Chengdu Sichuan Province China; ^3^ Department of Pulmonary and Critical Care Medicine West China Hospital, Sichuan University Chengdu China

**Keywords:** lung abscess, metagenomic next‐generation sequencing (mNGS), periodontitis, *Porphyromonas gingivalis*

## Abstract

Lung abscess is a common disease in respiratory medicine, which is a suppurative lesion caused by various pathogens, and microbiological examination is crucial for the treatment of lung abscess. Due to the widespread use of antibiotics, it is difficult to obtain reliable microbiological evidence through routine tests. There are various pathogens present in the oral cavity, and periodontitis is a risk factor for the formation of lung abscess. Enhancing understanding of lung abscesses caused by *Porphyromonas gingivalis* and the importance of accurately interpreting NGS reports. This article will present a case report of a lung abscess related to oral bacteria ( *Porphyromonas gingivalis* ). The patient was initially treated with empirical anti‐infective therapy, which was ineffective, and despite multiple sputum cultures and bronchoalveolar lavage fluid analysis using metagenomic next‐generation sequencing (mNGS), the pathogen could not be identified clearly. However, based on the significant presence of oral bacteria in the NGS of the bronchoalveolar lavage fluid, which guided the examination to discover periodontitis. Subsequently, a percutaneous lung tissue biopsy was performed for NGS testing, which further suggested *Porphyromonas gingivalis* as the pathogenic bacterium. This article summarizes the clinical manifestations, imaging findings, and characteristics of the pathogenic microorganisms in this case of lung abscess, reviews relevant literature to enhance the understanding of lung abscess caused by *Porphyromonas gingivalis* . It also confirms the importance of careful analysis of background bacteria in bronchoalveolar lavage fluid NGS based on objective risk factors, and highlights that combining patient clinical features with multisample NGS examination can promptly clarify the microbiology.

## Graphical abstract

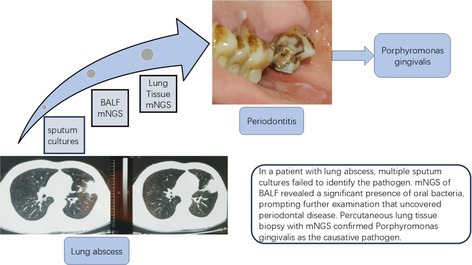

## 1 Introduction

Lung abscess is a localized infectious cavity in the lung tissue caused by viruses, bacteria, fungi, or parasitic pathogens. It is a necrotic cavitary lesion in the lung parenchyma, filled with pus and necrotic tissue [1]. Lung abscesses are often secondary to aspiration pneumonia [2]. Research reports indicate that risk factors for lung abscess include structural lung disease, poor oral hygiene, ventilator‐associated pneumonia, and alcoholism [3]. Currently, clinical manifestations combined with chest CT are still an important method for diagnosing lung abscess, although identifying pathogens is often challenging. A study by Maitre et al. reported that the positive rate of sputum cultures is only around 50% [4], while metagenomic next‐generation sequencing (NGS) provides a potential means for identifying pathogens in culture‐negative cases [5]. Correctly understanding and analyzing the main pathogenic bacteria identified by NGS is crucial for clinical treatment. Currently, there are few reports on lung abscesses caused by *Porphyromonas gingivalis* infection. This article summarizes the diagnosis and treatment process of a patient with *Porphyromonas gingivalis* –associated lung abscess admitted to the Department of Pulmonary and Critical Care Medicine at West China Hospital, Sichuan University, and reviews related literature. It aims to reveal the relationship and potential mechanisms between oral bacteria and lung abscesses, providing new insights for the clinical diagnosis and treatment of respiratory infectious diseases complicated by periodontitis.

## 2 Case Report

**FIGURE 1:**
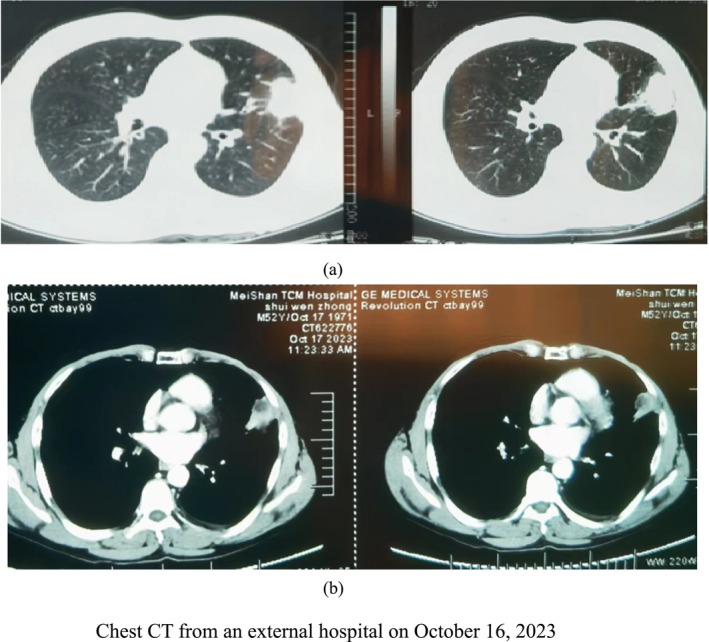
Chest CT from an external hospital on October 16, 2023.

**FIGURE 2:**
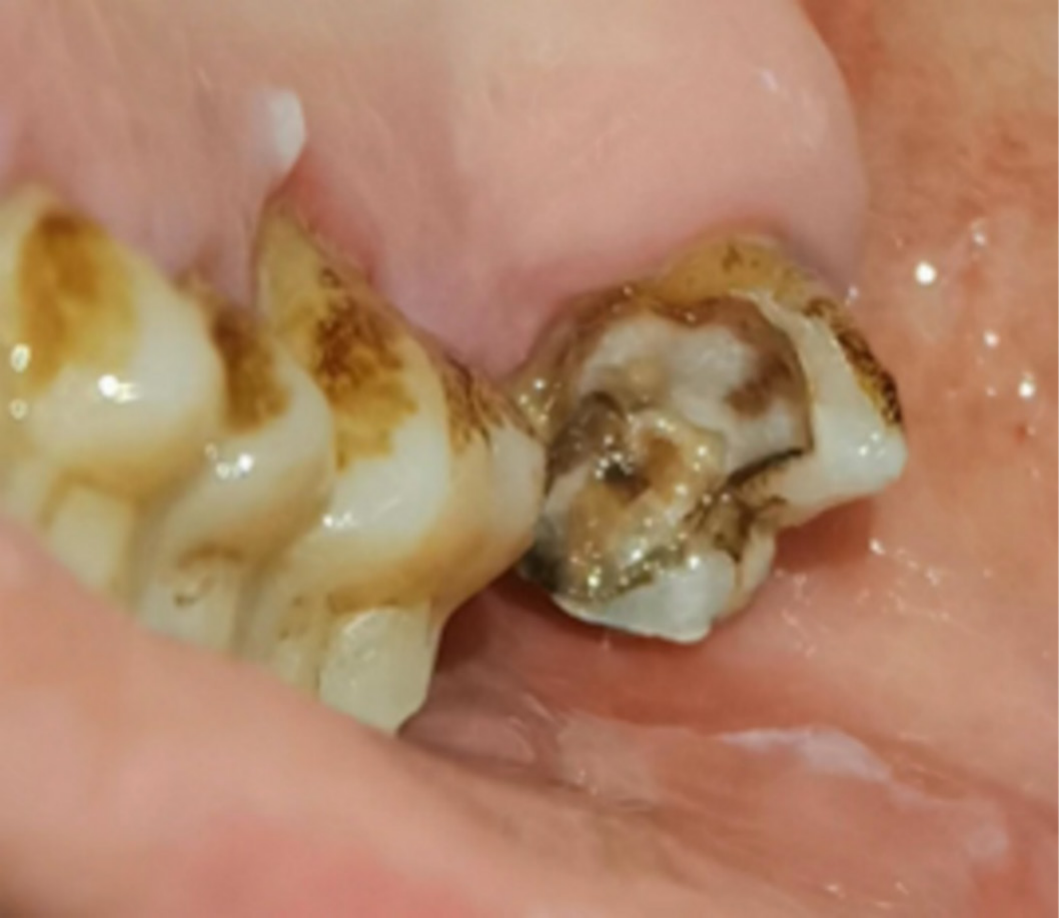
The patient's oral examination indicated the presence of periodontitis and dental caries.

The patient is a 52‐year‐old male, who was admitted on October 23, 2023, due to “cough, sputum production, and chest pain for one week, worsened with shortness of breath for one day.” One week prior, he experienced a cough and sputum production without any obvious inducement, accompanied by pulling pain in the left chest, but had no fever, hemoptysis, or orthopnea. He sought treatment at a local hospital where chest enhanced CT revealed an irregular soft tissue mass in the lingular segment of the left upper lobe (size approximately 4.5 cm × 3 cm), inflammatory changes in the right lower lobe, and multiple small micronodules in both lungs (Figure 1a,b). Laboratory tests indicated leukocyte count (WBC) of 14.06 × 10^9^/L, with a neutrophil percentage (NEU%) of 78.4%. The diagnosis was “lung abscess,” but treatment with ceftazidime was ineffective. One day prior to admission, the patient experienced shortness of breath and sought emergency care at our hospital. Enhanced chest CT: scattered patchy and striated shadows in both lungs, with partial consolidation, an area of patchy uneven enhancement in the left lung, small to moderate pleural effusion in the left thoracic cavity, atelectasis of adjacent lung tissue, and visible mediastinal lymph nodes, as well as thickening of the right pleura (Figure 2a,b). He underwent thoracentesis to extract approximately150ml of yellow turbid fluid and was subsequently admitted to our department. The patient has a 7‐year history of facial paralysis, treated with acupuncture, leaving residual involuntary facial muscle twitching. He has no history of hypertension, diabetes, coronary heart disease, or other significant medical conditions. He has a 30‐year smoking history (20 cigarettes per day), having quit 1 week prior; and a 30‐year history of drinking, averaging about 250 g daily, which he also quit 1 week ago.

On examination upon admission: temperature 37.5°C, pulse 96 bpm, respiration 22 bpm, blood pressure 126/77 mmHg, fingertip oxygen saturation 96% (with nasal cannula oxygen at 3 L/min). (SPO2:89% when the patient is not on oxygen). The chest wall appeared normal; left lung base percussion was dull, with diminished breath sounds, and no obvious dry or moist rales were auscultated in both lungs. Heart sounds normal and rhythmic, with no murmurs in the cardiac valve areas. The abdomen was soft, with no tenderness or rebound tenderness, and no edema in both lower limbs. Relevant auxiliary examinations: Emergency lab results indicated WBC 14.06 × 10^9^/L, hemoglobin 116 g/L, platelet count 336 × 10^9^/L, neutrophil percentage 94.8%, and lymphocyte absolute count 0.25 × 10^9^/L. Procalcitonin was 0.14 ng/mL. The pleural fluid analysis showed yellow, cloudy fluid, with nucleated cells 1788, erythrocytes 10 488, no pus cells, 56% were mononuclear cells, and 44% were polymorphonuclear cells. Biochemistry of the pleural fluid revealed total protein 47.9 g/L, lactate dehydrogenase (LDH) 890 IU/L, and glucose 8.76 mmol/L. Blood gas analysis (with nasal cannula oxygen at 3 L/min) showed: pH 7.43, PaO_2_ 80.8 mmHg, PaCO_2_ 37.9 mmHg, and HCO_3_^−^ 25 mmol/L. Cardiac enzymes, liver function, and renal function tests were normal. After admission, results for nasopharyngeal swab respiratory pathogen nucleic acid tests, serum Legionella antibody tests, serum *Cryptococcus neoformans* capsular antigen tests, serum *Chlamydia* and *Mycoplasma* antibody tests, 1,3‐β‐d‐glucan assay, *Aspergillus* galactomannan assay, serum *Aspergillus* IgG antibodies, serum lung tumor markers, pleural fluid acid‐fast staining, and TB‐Xpert were all negative. Pleural fluid pathology indicated a significant number of lymphocytes. Cultures of sputum and bronchoalveolar lavage fluid yielded few colonies of *Candida albicans* . Bronchoscopy and lavage results: Bronchoscopy showed no abnormalities in the airways. Bronchoalveolar lavage fluid was collected from the left lower lung lobe for testing. Pleural fluid, bronchial washing fluid, and transbronchial lung biopsy samples were all cultured for Nocardia, acid‐fast bacilli, and aerobic/anaerobic bacteria, with all results being negative. Metagenomic next‐generation sequencing (mNGS) of the bronchoalveolar lavage fluid revealed: *Candida albicans* (sequence count: 255; relative abundance: 25.68%), human gamma herpesvirus 4 (EBV) (sequence count: 31; relative abundance: 65.00%), human beta herpesvirus 5 (CMV) (sequence count: 25; relative abundance: 35.00%), suspected background organisms list: *Prevotella salivae* (sequence count: 1102) *Streptococcus peroris* (sequence count:138), *Veillonella atypica* (sequence count: 362), *Porphyromonas gingivalis* (sequence count: 192), *Abiotrophia defectiva* (sequence count: 363), *Neisseria elongata* (sequence count: 48), *Megasphaera micronuciformis* (sequence count: 68), *Atopobium parvulum* (sequence count: 66), *Actinomyces graevenitzii* (sequence count: 26). No microbes were detected in the pleural fluid NGS results. External hospital percutaneous lung biopsy NGS results: *Porphyromonas gingivalis* (sequence count: 1243; relative abundance: 69.89%). All samples were submitted to the Precision Medicine Center—Department of Experimental Medicine at West China Hospital, Sichuan University, for PMseq high‐throughput pathogen gene testing. This method is based on metagenomic technology, which extracts nucleic acids from all microorganisms in the infected samples, followed by different sample preprocessing. Sequencing was conducted on the BGI high‐throughput sequencing platform at the Precision Medicine Center of West China Hospital, Sichuan University. Through comparison with the high‐quality and high‐standard clinical‐grade database PMDB and using various patented intelligent bioinformatics analysis algorithms, we obtained taxonomic information on suspected pathogenic microorganisms. This unbiased, one‐time test can detect 17 500 pathogens including bacteria, fungi, viruses, and parasites, significantly improving the positive rate of pathogen diagnosis, guiding targeted antibiotic use in clinical practice, and assisting in the precise diagnosis and treatment of infections.

**FIGURE 3:**
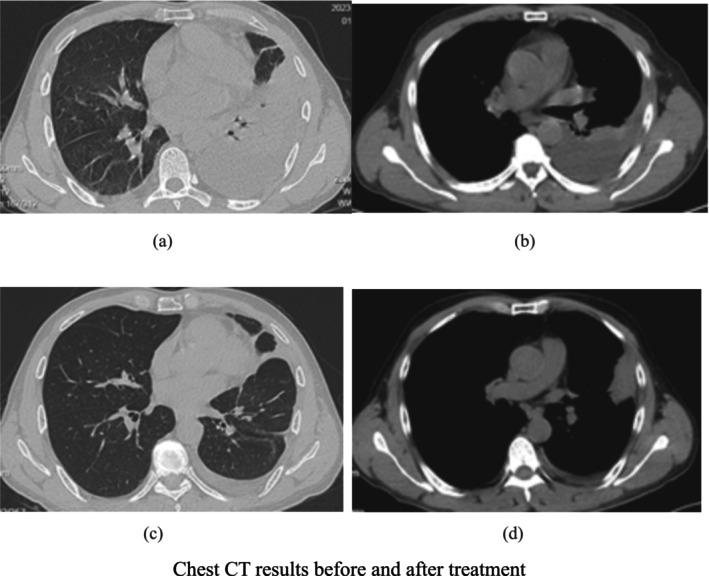
Chest CT results before and after treatment. (a,b) Emergency chest CT on October 22, 2023. (c,d) Chest CT from the Department of Respiratory and Critical Care Medicine on November 2, 2023.

Treatment Process: After being admitted on October 23, 2023, the patient was given oxygen therapy and treated with moxifloxacin (0.4 g, once daily, intravenous infusion) combined with meropenem (1 g, every 8 h, intravenous infusion) for infection control, along with expectorants and nebulization. However, the patient developed worsening cough, shortness of breath, chest pain, fever, and purulent hemoptysis. Upon careful analysis of the bronchoalveolar lavage fluid and pleural fluid NGS results, and after tracing the patient's long history of poor oral hygiene, a detailed physical examination revealed that the patient had dental caries and periodontitis (Figure 2). A consideration of a dental source of bacterial infection led to an adjustment of antibiotics on October 29, 2023, to penicillin (4 million units, every 6 h, intravenous infusion) and metronidazole (0.4 g, once daily, intravenous infusion). After 4 days of treatment, the patient no longer experienced fever or hemoptysis, and there was significant relief of cough, sputum production, chest pain, and dyspnea. A follow‐up blood test showed normal complete blood count and PCT levels. On November 2, 2023, a repeat chest CT scan showed a reduction in the size of the infection site, with partial absorption (Figure 3c,d). The patient's condition improved, and they were discharged on November 3, 2023.

**FIGURE 4:**
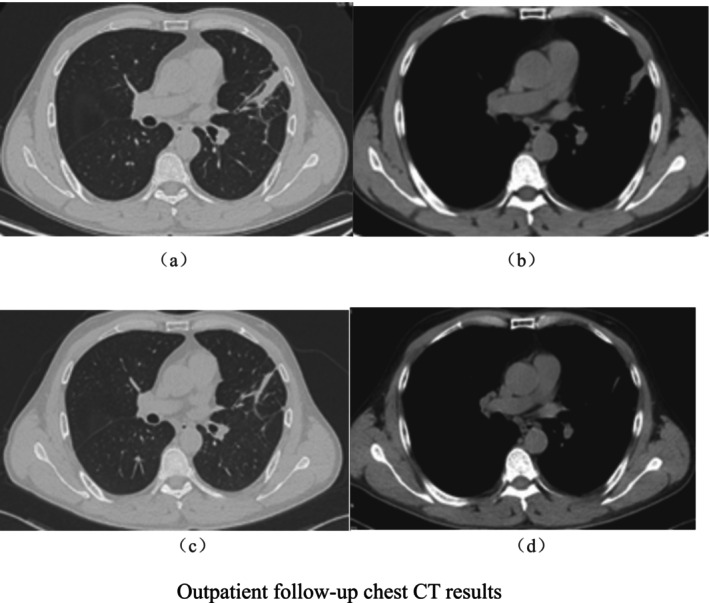
Outpatient follow‐up chest CT results. (a,b) Outpatient chest CT re‐examination on November 27, 2023. (c,d) Outpatient chest CT re‐examination on January 10, 2024.

Follow‐up and Outcome: After discharge, the patient was prescribed long‐term oral amoxicillin‐clavulanate (1 g twice daily) and metronidazole (200 mg three times daily). Vital signs remained stable, and respiratory symptoms significantly improved. On November 27, 2023, a follow‐up chest CT showed scattered mild inflammation in both lungs and a band‐like soft tissue shadow subpleural in the left upper lobe, mostly infected. Compared with the CT from November 2, 2023, the lesions had noticeably decreased in size, and inflammation in both lungs had reduced. On January 10, 2024, a subsequent outpatient chest CT revealed a subpleural strip of soft tissue in the left upper lobe, indicating mostly infected, which had slightly diminished compared with the previous examination. A follow‐up chest CT on January 10, 2024, in the outpatient clinic showed a band‐like soft tissue shadow subpleural in the left upper lobe, which was slightly smaller than before, little chronic inflammation in both lungs, little pleural effusion on the left side, and left lung re‐expansion (Figure 4).

## 3 Literature Search

Articles containing keywords such as “lung abscess,” “pulmonary infection,” “respiratory tract infection,” “ *Porphyromonas gingivalis* ,” and “oral pathogens” were retrieved from the Web of Science, PubMed, and Wanfang Data databases. Relevant articles related to respiratory tract infections and oral pathogens, including reviews, case reports, and randomized controlled trials (RCTs), were selected. Literature unrelated to the topic, conference abstracts, and articles without full‐text availability were excluded. The most relevant references aligned with the theme are listed in Table 1, while case report articles are shown in Table 2.

**TABLE 1:** Related studies.

Study	Year	Study design	Methods	Materials	Results
Potential role of periodontal infection in respiratory diseases—A review [6]	2013	Review			Periodontal disease, may influence the course of respiratory infections. The mechanisms of infection could be the aspiration into the lung of oral pathogens capable of causing pneumonia, colonization of dental plaque by respiratory pathogens followed by aspiration, or facilitation of colonization of the upper airway by pulmonary pathogens by periodontal pathogens.
A pulmonary abscess caused by *Porphyromonas endodontalis* infection: A case report and literature review [7]	2024	Case report	mNGS	1	Following mNGS analysis of the liquefaction necrotic area and solid component of the lesion through biopsy, *Porphyromonas endodontalis* and *Parvimonas micra* were detected.
Subcutaneous abscess due to empyema necessitans caused by *Porphyromonas gingivalis* in a patient with periodontitis [8]	2022	Case report	Cultivation	1	The puncture‐aspirated pus from the subcutaneous abscess yielded a pure culture of *P. gingivalis*
Pyopneumothorax with bronchopleural fistula due to pulmonary infection caused by *Porphyromonas gingivalis* in a patient with periodontitis [9]	2023	Case report	mNGS and anaerobic culture.	1	*P. gingivalis* positive
Significance of anaerobes and oral bacteria in community‐acquired pneumonia [10]	2013	A case–control study	mNGS, conventional cultivation methods, including anaerobic cultures	64	The incidence of anaerobes and oral bacteria in CAP patients, especially in patients with mild PSI, is higher than previously reported. These bacteria may play important roles in CAP.
Periodontal infections and community‐acquired pneumonia: a case–control study [11]	2013	A case–control study	A periodontal examination	140	Chronic periodontitis (CP) was more frequent in patients with CAP, The presence of moderate or severe CP increased the risk for CAP, Moderate and severe chronic periodontitis were associated with CAP in this study.
Influence of periodontitis in the development of nosocomial pneumonia: a case control study [12]	2014	A case–control study	Physical, microbiologic, and/or radiographic examination	315	Individuals with periodontitis were three times as likely to present with NP. Periodontal infection may influence the development of NP
Potential associations between chronic respiratory disease and periodontal disease: analysis of National Health and Nutrition Examination Survey III [13]	2001	Cross‐sectional, retrospective study	Lung function, radiographic examination	13 792	Subjects with a history of COPD had more periodontal attachment loss than subjects without COPD. A trend was noted in that lung function appeared to diminish with increasing periodontal attachment loss. The findings of the present analysis support recently published reports that suggest an association between periodontal disease and COPD.
Prevalencia de enfermedad periodontal grave (EPG) y su asociación con enfermedades respiratorias en pacientes adultos hospitalizados en un centro de tercer nivel (Prevalence of severe periodontal disease and its association with respiratory disease in hospitalized adult patients in a tertiary care center) [14]	2015	Cross‐sectional study	A periodontal examination, radiographic examination	359	High prevalence of severe periodontal disease was observed in the different respiratory diseases. Severe periodontal disease was associated with both infectious and noninfectious respiratory diseases. It is important to study an oral health intervention.
Periodontopathogens *Porphyromonas gingivalis* and *Fusobacterium nucleatum* and Their Roles in the Progression of Respiratory Diseases [15]	2023	Review			The progression of related respiratory diseases are summarized and analyzed, including: induction of mucus hypersecretion and chronic airway inflammation; cytotoxic effects to disrupt the morphology and function of respiratory epithelial cells; synergistic pathogenic effects with respiratory pathogens like *Streptococcus pneumoniae* and *Pseudomonas aeruginosa*
Relationship between the oral cavity and respiratory diseases: Aspiration of oral bacteria possibly contributes to the progression of lower airway inflammation [16]	2021	Review			Clarifying these mechanisms that chronic periodontitis and oral bacteria contribute to lower airway diseases. Relationship between oral cavity and respiratory diseases: “Oral bacterium–virus–host interactions” in the oral cavity–lower airway axis.
Use of metagenomic next‐generation sequencing in diagnosing lung abscesses caused by oral bacteria [17]	2023	Case report	mNGS	1	A lung abscess caused by oral bacteria
A higher significance of anaerobes: the clone library analysis of bacterial pleurisy [18]	2011	Prospective observational study	Conventional cultivation, NGS	42	The clone library analysis using the 16S rDNA of pleural fluid showed a higher incidence of anaerobic bacteria in infectious pleurisy than that previously expected and provided additional bacterial information for cultivation methods.

**TABLE 2:** Related case reports.

Study	Year	Study design	Methods	Materials	Results
A pulmonary abscess caused by *Porphyromonas endodontalis* infection: A case report and literature review [7]	2024	Case report	mNGS	One patient, the liquefaction necrotic area and solid component of the lesion	Following mNGS analysis of the liquefaction necrotic area and solid component of the lesion through biopsy, *Porphyromonas endodontalis* and *Parvimonas micra* were detected.
Subcutaneous abscess due to empyema necessitans caused by *Porphyromonas gingivalis* in a patient with periodontitis [8]	2022	Case report	Cultivation	One patient, puncture—aspirated pus from the subcutaneous abscess	The puncture‐aspirated pus from the subcutaneous abscess yielded a pure culture of *P. gingivalis*
Pyopneumothorax with bronchopleural fistula due to pulmonary infection caused by *P. gingivalis* in a patient with periodontitis [9]	2023	Case report	mNGS and anaerobic culture.	One patient. Pleural puncture yieldedpus and peripheral blood	*P. gingivalis* positive
Use of metagenomic next‐generation sequencing in diagnosing lung abscesses caused by oral bacteria [17]	2023	Case report	mNGS	One patient. Pleural fluid	A lung abscess caused by oral bacteria

## 4 Discussion

Lung abscesses are common infectious diseases of the lower respiratory tract, and many pathogens are derived from the oral cavity. *Porphyromonas gingivalis* is a Gram‐negative anaerobic bacterium that long resides in the oral cavity and serves as a major pathogen for chronic periodontitis [19]. *Porphyromonas gingivalis* typically exists in the form of cocci or rod‐shaped bacteria. It can produce collagenase, a series of proteases, hemolysin, endotoxins, fatty acids, ammonia, hydrogen sulfide, indole, and other substances. It is able to attack epithelial cells of the gingival mucosa and endothelial cells [20]. There are relatively few reports in the literature regarding lung abscesses caused by *Porphyromonas gingivalis* . A systematic review published by Bansal et al. in 2013 suggested that *Porphyromonas gingivalis* , can cause pneumonia and lung abscesses [6]. Li et al. reported a case of a lung abscess caused by *Porphyromonas gingivalis* in 2023 [7]. Tanaka et al. reported a case in 2022 where a patient with periodontitis developed a subcutaneous abscess due to *Porphyromonas gingivalis* causing empyema [8]. Sha et al. reported in 2023 a case of septic pneumothorax with bronchopleural fistula due to pulmonary infection caused by *Porphyromonas gingivalis* in a patient with periodontitis [9]. Reports indicate that 86% of subgingival plaque samples from patients with periodontitis contain *Porphyromonas gingivalis* [10]. Dental plaque releases bacteria into saliva, which may be inhaled into the lower respiratory tract, thereby facilitating the onset and progression of pulmonary infections [6]. For this patient, the initial etiology was unclear, and after routine anti‐infection treatment, the patient's condition worsened. The mNGS analysis of the bronchoalveolar lavage fluid indicated a significant presence of dental‐origin bacteria, such as Prevotella species, *Streptococcus anginosus* , *Porphyromonas gingivalis* , and *Actinomyces graevenitzii* . A further inquiry into the patient's history revealed periodontitis, and an oral examination identified molars with exposed pulp, suggesting a possible dental source of bacterial infection. Additionally, mNGS testing of a percutaneous lung biopsy specimen detected a high number of sequences for *Porphyromonas gingivalis* , which is not a commensal organism of lung tissue. Therefore, it was ultimately concluded that *Porphyromonas gingivalis* was the pathogenic bacterium responsible for the lung abscess.

The oral cavity represents the second‐largest microbial community in humans, hosting over 700 types of microorganisms, including bacteria, fungi, protozoa, mycoplasma, and viruses [21]. In healthy individuals, the highest bacterial density in normal saliva is 10^8^ CFUs/mL; therefore, it is presumed that individuals with poor oral hygiene have a bacterial load in their saliva exceeding 10^8^ CFUs/mL [22]. The oral cavity and the lower respiratory tract are connected, allowing bacteria to enter the lower airways from the mouth; thus, the oral cavity is also a major pathway for the lung microbiota [23]. When a substantial number of microorganisms enter the lower respiratory tract beyond the clearing ability of the defense system or when host immunity is compromised, the weakened defense system may fail to clear the inhaled microbes in a timely manner, allowing oral microbes to cause respiratory infections [24, 25]. Recently, many studies have reported that chronic periodontitis and oral bacteria are associated with the development, progression, and worsening of several respiratory diseases. de Melo Neto et al. found that patients with moderate to severe chronic periodontitis have a 4.4‐fold increased risk of developing community‐acquired pneumonia compared with nonperiodontitis patients [11]. Gomes‐Filho et al. discovered that patients with moderate to severe chronic periodontitis had a 2.9‐fold higher risk of developing hospital‐acquired pneumonia compared with nonperiodontitis patients [12]. Scannapieco proposed that among smokers, patients with severe chronic periodontitis have a 3.71‐fold increased risk of developing chronic obstructive pulmonary disease (COPD) compared with those without periodontitis [13]. Fernández‐Plata et al. conducted a cross‐sectional study involving 3059 patients and found that the probability of developing lung abscesses in patients with severe periodontal disease is 2.6 times higher than in patients without periodontal disease [14]. Therefore, considering this patient, the impact of oral health on lung diseases should be taken seriously.

*Porphyromonas gingivalis* exhibits its pathogenicity primarily through the following mechanisms [15]: (1) adhesion and invasion capabilities mediated by fimbriae, hemagglutinin, and proteins; (2) induction of peripheral blood CD4^+^ T helper cells to produce pro‐inflammatory cytokines, such as IL‐1 and IL‐6; (3) activation of T lymphocyte immune responses, promoting receptor activator of nuclear factor‐κB ligand (RANKL)‐induced osteoclast activation; (4) diminished chemokine response induced by dendritic cells; (5) production and secretion of gingival proteases, such as arginine‐ and lysine‐specific cysteine proteases; (6) secretion of outer membrane vesicles (OMVs) to deliver virulence factors. Imai et al. studied the mechanisms through which oral bacteria exacerbate lower airway inflammation [16]: when poor oral hygiene leads to the aspiration of oral bacteria, including periodontal pathogens, lower airway epithelial cells express more platelet‐activating factor receptors (PAFRs), which are receptors for bacteria that induce lung inflammation. These epithelial cells also secrete inflammatory cytokines. Moreover, the aspiration of oral bacteria can induce bronchial glands to produce excessive mucus, leading to bronchial lumen obstruction and decreased respiratory function, as well as damaging alveolar and bronchial epithelial barriers, allowing viruses and other microbes to penetrate subepithelially. Additionally, inflammatory substances produced in the oral tissues can influence systemic organs, including the lungs, through the bloodstream (the impact of chronic periodontitis on systemic chronic inflammation), and the aspiration of oral bacteria may cause dysbiosis in the lungs (imbalance in local pulmonary microbiota). Through these mechanisms, “chronic periodontitis and oral bacteria” may contribute to the occurrence and progression of lower airway diseases. This highlights the significant role of oral health management in preventing the onset and progression of lower respiratory tract diseases. In the case of this patient, long‐term poor brushing habits, dental caries, and periodontitis led to a high presence of *Porphyromonas gingivalis* in the oral cavity, thus participating in the pathogenesis of lung abscesses through the aforementioned mechanisms. Treatment of *Porphyromonas gingivalis* infections primarily involves robust antibiotic therapy. However, the widespread use of antibiotics has led to emerging resistance. In research conducted by Ardila et al., resistance rates were found to be 24.6%, 21.3%, and 24.6% for amoxicillin, azithromycin, and metronidazole, respectively [26]. However, in this case, the patient was treated sequentially with penicillin and metronidazole via intravenous infusion, which quickly controlled the condition. This indicates that the *Porphyromonas gingivalis* in this patient was non‐resistant. Nevertheless, we should remain vigilant for the emergence of resistant bacteria in similar patients and prepare appropriate alternative treatment options.

Pulmonary abscesses can progress rapidly and may pose a significant threat to a patient's life. Therefore, quickly establishing a microbiological diagnosis and employing an antibiotic treatment plan that can cover the pathogenic bacteria is crucial. Using sputum specimens to identify pathogens carries the risk of contamination from oral flora, and the positive rate is not high [3]. Metagenomic next‐generation sequencing (mNGS) utilizing reliable specimens can aid in establishing the microbiological diagnosis of pulmonary abscesses, distinguishing infection from colonization, and guiding treatment [17]. mNGS is a novel clinical metagenomic technology based on sequencing that does not require culture. By analyzing the quantity and abundance of microbial DNA or RNA in clinical samples, pathogens can be identified [27]. Conventional microbiological culture remains the preferred method for diagnosis; however, for uncommon pathogens (such as *Mycobacterium tuberculosis* , fungi, viruses, and anaerobes), mNGS may be more effective than microbial culture [18, 28]. In this case, after initial empiric anti‐infection treatment, the patient's condition worsened, and the pathogen was unknown, with both sputum and pleural fluid microbiological tests returning negative results. Bronchoscopic lavage fluid NGS only detected *Candida albicans* , which did not align with the clinical presentation and imaging, creating a dilemma in identifying the pathogen. Upon carefully analyzing the NGS results of the bronchoalveolar lavage fluid, we discovered a significant presence of oral bacteria among the background flora. This prompted a closer clinical examination, revealing that the patient had periodontal disease and poor oral hygiene. Subsequently, a transcutaneous lung biopsy was performed using NGS, which ultimately identified a high sequence count of *Porphyromonas gingivalis* . This targeted treatment led to a rapid recovery from the disease. This case illustrates the critical role of accurately interpreting NGS reports in clinical settings. To effectively interpret NGS reports, one must understand the NGS testing technology. NGS reports are the technical interpretation results obtained by laboratory technicians and bioinformatics analysts comparing data [29]. There are many different NGS testing institutions on the market, and their reporting principles generally include: they will definitely report clearly pathogenic bacteria, even if the sequence count is low, such as Legionella and *Chlamydia psittaci* ; for background bacteria, the report will retain some conditionally pathogenic bacteria that are controversial (suspected background microorganisms), while others will be excluded. Thus, these subjective factors may lead to inaccurate results. Suspected background microorganisms refer to normal symbiotic/colonizing microbes present in the human body, and their potential to cause infection cannot be excluded. The distinction between colonization and infection often relies on clinical experience and conclusions from databases regarding potential colonizers [30]. The 2023 expert consensus on the clinical interpretation pathway for metagenomic next‐generation sequencing of lower respiratory tract infections states that in patients with a risk of aspiration or a clear history of aspiration, the presence of various common oral colonizing bacteria in NGS results should prompt consideration of aspiration pneumonia/lung abscess [31]. Although the positive rate of mNGS testing from transcutaneous lung biopsy specimens is not higher than that from BALF, the detection results from transcutaneous lung biopsy specimens can corroborate and complement the findings from BALF for peripheral lung lesions [31]. Therefore, in this case of a lung abscess with a high presence of oral colonizing bacteria, the combination of NGS testing from bronchoalveolar lavage fluid and transcutaneous lung biopsy tissue ultimately identified *Porphyromonas gingivalis* as the pathogenic bacterium.

In conclusion, effective and rapid pathogen identification is crucial in the diagnosis and treatment of lower respiratory tract infections. This case analysis highlights the clinical significance of oral bacteria in causing pulmonary infectious diseases. Timely and decisive implementation of diverse sample mNGS testing, along with comprehensive and careful analysis of the results, was key to the successful treatment of this case. It is important to note that when interpreting mNGS reports, clinical context must be considered, and one should not overlook background bacteria, as they may carry important implications.

Through this case report and literature review, it is emphasized that especially for patients at risk of aspiration, oral hygiene and dental health should be prioritized. Moving forward, the interpretation of pathogen detection via NGS in patients should not focus solely on pathogenic bacteria; instead, it should be combined with clinical data. Additionally, greater efforts should be made to strengthen basic research on *Porphyromonas gingivalis* , optimize NGS testing methods, and improve the diagnostic positive rate for lung infections related to alveolar bacteria.

## Author Contributions

**Xu Chen**: formal analysis, writing – original draft, writing – review and editing. **Ling Wu**: formal analysis, writing – original draft, writing – review and editing. **Ruoxi Wu**: writing – review and editing. **Jiajia Dong**: writing – review and editing.

## Ethics Statement

This case report has been approved by the Ethics Committee of West China Hospital, Sichuan University. In this study, the patient gave written informed consent for his personal or clinical details, along with any identifying images, to be published.

## Conflicts of Interest

The authors declare no conflicts of interest.

## Data Availability

The data that support the findings of this study are available on request from the corresponding author. The data are not publicly available due to privacy or ethical restrictions.
